# Soil viral communities shifted significantly after wildfire in chaparral and woodland habitats

**DOI:** 10.1093/ismeco/ycaf073

**Published:** 2025-05-06

**Authors:** Sara E Geonczy, Anneliek M ter Horst, Joanne B Emerson

**Affiliations:** Department of Plant Pathology, University of California, Davis, One Shields Avenue, Davis, CA 95616, United States; Department of Plant Pathology, University of California, Davis, One Shields Avenue, Davis, CA 95616, United States; Department of Plant Pathology, University of California, Davis, One Shields Avenue, Davis, CA 95616, United States

**Keywords:** soil viral ecology, viromics, fire, wildfire, chaparral, woodland

## Abstract

Increased wildfire activity warrants more research into fire-driven biotic changes in soil, including soil viral communities, given the roles of soil microbes in organic matter decomposition, nutrient cycling, and post-fire recovery. Leveraging viral size-fraction metagenomes (viromes), here we studied viral community responses to wildfire in woodland and chaparral soils at five timepoints over 1 year following the California LNU Complex wildfire. We also compared post-fire samples to unburned controls at the final three timepoints and leveraged published viromes from the same sites nine months before the fire as pre-burn controls. Viral community composition differed significantly in burned samples compared to controls from both habitats, as did soil chemistry and prokaryotic communities (16S rRNA gene amplicons). Viromic DNA yields (a proxy for viral particle abundances) indicated initial viral biomass reductions due to the fire, but a return to baseline abundances (indistinguishable from controls) within five months. Fire-associated habitat filtering was further indicated by a comparison to the PIGEON viral “species” (viral operational taxonomic unit (vOTU)) reference database, with vOTUs from a burned conifer forest representing 19%–31% of PIGEON vOTUs detected in the burned habitats but only 0.6%–6% in controls. Together, these results indicate significant changes in soil viral communities due to wildfire, attributable at least in part to concomitant changes in their prokaryotic host communities and soil physicochemistry.

## Introduction

The current increase in global fire activity, driven by climate change, land use change, and fire suppression policies, warrants a better understanding of fire’s effects on biogeochemical processes and ecosystem resiliency in Mediterranean shrubland and woodland habitats [1–3]. This includes the impact on prokaryotic communities, which are significant drivers of soil biogeochemical processes, with major roles in organic matter decomposition and nutrient cycling [4, 5]. Changes in soil properties, which often occur after a disturbance like a wildfire [6], are well known to drive bacterial community shifts [7]. One indirect impact of fire is a decrease in soil moisture, which can impact microbial communities, especially in habitats with low precipitation already [8]. Soil viruses, an understudied component of the soil microbiome, infect prokaryotes and are also known to be impacted by drought and heat [9, 10], but the short- and long-term impacts of wildfire on soil viral communities and the roles that viruses may play in the post-fire environment are largely unknown.

Wildfires can transform landscapes and sometimes act as an ecosystem “reset”, driving primary and secondary plant succession [11]. Some habitats are wildfire-mediated, including chaparral, with vegetation that evolved with fire that has adaptations both conducive and sometimes dependent on a certain frequency of fire activity [12]. Soil bacterial community shifts can influence ecosystem successional rates [13, 14], which makes post-fire soil microbial dynamics of great interest, given the threat of loss of habitat after wildfires [13, 15]. Studies with high-resolution temporal sampling after fire have shown rapid bacterial community change, including both dramatic shifts immediately after fire and frequent community compositional changes over time [12]. For example, pyrophilous bacteria in soil, such as *Massilia* (Proteobacteria phylum), *Flavobacterium* (Bacteroidota phylum), *Arthrobacter* (Actinobacteriota phylum), and *Bacillus* (Firmicutes phylum) have been identified for their proliferation after fire, which has been attributed to traits that confer success in a post-fire environment, such as fast growth, heat tolerance, xerotolerance, spore formation, and degradation of complex carbon compounds in pyrolyzed organic material [12, 16–20]. Additionally, at least some pyrophilous bacteria seem to be phylogenetically conserved, with fire selecting for increased relative abundances of Firmicutes and Actinobacteria, which may mean that these taxa are both adapted to and ubiquitous across habitats that have experienced a fire disturbance [20]. Although heat-responsive viral “species” (viral operational taxonomic units (vOTUs)) have been identified [10], the extent to which there might be “pyrophilous” soil viruses is unknown.

While bacterial succession after wildfire has been studied in chaparral [12], little is known about the communities in woodland habitats that are often found close to chaparral. Additionally, little is known about the viruses in these same systems in general [21], especially with regard to the post-fire response. Here we present a 1-year temporal study on viral and prokaryotic communities in woodland and chaparral soils following a wildfire. Our aim was to compare responses between the two habitats over time while also comparing community responses to dynamics in non-fire-impacted controls and to viromes previously collected from the same sites 9 months before the fire [21]. Additionally, we sought to place the viruses detected in disturbed and undisturbed samples in biogeographic context, assessing where they had been detected previously and whether the detection patterns might suggest conserved viral adaptations to fire.

## Materials and methods

### Study site description

The LNU Lightning Complex fires burned 147 000 ha in northern California and were active for 46 days from 17 August to 2 October 2020 [22]. On 4 November 2020, we set up four 5 × 5 m plots at Quail Ridge Natural Reserve, CA, which burned at low-to-moderate severity in all locations throughout the field site, determined via the Monitoring Trends in Burn Severity (MTBS) dataset [23]. Two plots were in burned chaparral habitats and two in burned woodland habitats. Plots were spatially paired, meaning that each chaparral plot was located close to (within 85 m of) a spatially paired woodland plot. These plots were all considered burned plots. On 12 March 2021, we set up an additional four burned plots (two chaparral and two woodland, which were spatially paired within 150 m of each other) at McLaughlin Natural Reserve, CA and four control plots (two chaparral and two woodland, which were not spatially paired due to the constraint of sampling locations that had not been burned by the wildfire). We also leveraged seven viromes from seven sites collected in November 2019 as part of a separate study [21], allowing us to make pre-burn comparisons for the Quail Ridge site, which burned completely in the LNU Complex fires. Overall, there were 19 sampling locations (Supplementary Table S1). Chaparral sites had loamy soil with minimal horizon development, with surface mineral soil (A horizon) extending down to 5–15 cm. Woodland sites had predominantly loamy clay-enriched soil with an A horizon down to 25 cm. There was no organic horizon at any of the sites [24]. Chamise was the dominant vegetation for all chaparral sites, and oak and pine trees were the dominant vegetation for woodland sites.

### Soil sampling

At each of the 12 plots set up post-fire, we collected two replicates per time point in each plot by dividing the plot in half along the middle, then dividing the subplot into eight sampling units, and collecting soil (0–6 cm depth) in corresponding sampling units at each timepoint (e.g. the top right of the right subplot and the top right of the left subplot for a single timepoint). The distance between replicate samples within each plot was ~3.75 m. We sampled five timepoints at each of the four plots at Quail Ridge (T1: 4 November 2020, 33 days since fire, T2: 13 January 2021, 103 days since fire, T3: 12 March 2021, 161 days since fire, T4: 17 May 2021, 227 days since fire, and T5: 9 August 2021, 311 days since fire). This yielded eight samples per timepoint, for a total of 40 samples. Additionally, we sampled three timepoints at each of the eight plots at McLaughlin (corresponding with the final three timepoints at Quail Ridge (T3–T5)) either on the same day or ± 1 day (16 samples per timepoint, for a total of 48 samples). McLaughlin samples (including burned and control samples) were not collected at T1 or T2 for two primary reasons: the site was too far away (more than a 2-h drive in each direction) for reasonable social distancing for COVID during the drive/during possible overnight stays, and frankly, we did not consider early enough that a site so far away could be a reasonable control (the ideal control would have been an unburned location at the same site as the T1–T2 burned plots, but the Quail Ridge field site burned in its entirety; there were no unburned locations available). We homogenized and sieved (8 mm) all samples separately. Samples were stored on ice and transported back to the lab and promptly placed at 4°C until DNA extraction two to three days after sample collection. Combined, 88 samples were collected as part of the post-fire component of this study across the two sites, adding to the seven samples collected pre-fire.

## Soil physicochemical measurements

During sample collection, in-field soil temperature measurements were taken at each sampling location, and water repellency was measured by wetting a sample with a single drop of deionized water and counting how many seconds it took until the drop had fully infiltrated into the soil [25] (Supplementary Table S2). For the remaining soil physicochemical measurements, we note that there were some methodological differences among time points, which we clarify in detail here. In particular, differences between T0 (from our prior work at the same site) and T1–T5 are in part on account of not anticipating a need to compare these datasets, as well as differences in protocols before (T0) and during (T1–T5) the COVID-19 pandemic. Differences within the T1-T5 dataset were largely for cost-effectiveness, as we learned in real time about costs and options at different testing facilities. For T1–T5, we calculated gravimetric soil moisture of each sample by weighing out 10 g of soil into an aluminum weigh dish and measuring the weight after 24 h in a drying oven at 105°C. For T0, gravimetric soil moisture was calculated by weighing out 10 g of soil and allowing it to dry completely in a biological hood for up to several days before weighing again. For T0 (from our prior study), soil samples were sent to Ward Laboratories (Kearney, NE, USA) for basic soil tests. For T1, we sent a soil sample from each sample site (at this point, only the eight samples from Quail Ridge) to A&L Western Agricultural Laboratories (Modesto, CA, USA) for basic soil chemistry analysis as well as the University of California Davis Analytical Lab (Davis, CA, USA) for additional soil chemistry analysis. For timepoints T2–T5, we homogenized subplot replicate soil samples and sent a representative 200 g of field-condition soil (not dried) to Ward Laboratories for a suite of soil chemistry analyses under the Haney Soil Health Analysis package [26, 27]. Analysis was done by using the soil properties that we have available for all timepoints, which includes: gravimetric soil moisture (difference in method described above, soil pH (1:1 soil/water suspension), organic matter (percentage weight loss-on-ignition), nitrate (T0–T1 was KCl-extracted and T2–T5 was Haney-extracted), inorganic phosphorus (Olsen method for T0–1 and Haney-extracted and quantified by flow injection analysis for T2–T5), and potassium, calcium, magnesium, and sodium (T0–T1 was ammonium-acetate extracted and T2–T5 was Haney-extracted). All Haney-extracted nutrients were quantified using inductively coupled argon plasma (ICAP) atomic emission spectroscopy (Supplementary Table S3).

### Viromic DNA extraction and quantification

Within two or three days after sample collection (12 samples were randomly processed each day), we extracted viromic DNA from all samples, as previously described [10, 28]. Briefly, to 10 g of each soil sample, we added 9 ml of protein-supplemented phosphate-buffered saline solution (PPBS: 2% bovine serum albumin, 10% phosphate-buffered saline, 1% potassium citrate, and 150 mM MgSO_4_, pH 6.5). Samples were vortexed until homogenized and then placed on a horizontal shaker at 300 rpm at 4°C for 10 min. Supernatant was transferred into a new tube, and then 9 ml of PPBS was added to the soil remaining in the original tube, repeating the process for a total of three soil resuspensions and elutions, ending with a total of three volumes of supernatant pooled together in a new centrifuge tube. The pooled supernatant was then centrifuged at 8 min at 7840 rpm at 4°C, collecting the progressively clearer supernatant and discarding the concentrated soil particles, for a total of three centrifugations and supernatant collections. The final supernatant was filtered through a 5 μm polyethersulfone (PES) membrane filter (PALL, Port Washington, NY, USA) to filter out larger soil particles, and then the filtrate went through a 0.22 μm PES membrane filter (PALL) to exclude most cellular organisms (apart from ultra-small bacteria [9, 29]). Viral-sized particles and DNA remaining in the filtrate were concentrated into a pellet using the Optima LE-80 K ultracentrifuge with a 50.2 Ti rotor (Beckman-Coulter, Brea, CA, USA) at 32 000 rpm at 4°C for 2 h 25 min. The pellet was resuspended in 200 μl of 0.02 μm filtered ultrapure water and treated with DNase (briefly, incubation at 37°C for 30 min after adding 10 units of RQ1 RNase-free DNase, Promega, Madison, WI, USA). We then extracted DNA using the DNeasy PowerSoil Pro kit (Qiagen, Hilden, Germany), following the manufacturer’s protocol, with the addition of a 10 min incubation at 65°C prior to cell lysis, which also served as the “stop” incubation for the DNase treatment. DNA quantification was performed using the Qubit dsDNA HS Assay and Qubit 4 fluorometer (ThermoFisher, Waltham, MA, USA). Due to low (30 viromes) or undetectable DNA yields (concentration below 0.005 ng/μl or 0.049 ng/g soil) (19 viromes) now known to be expected for dry, heated, and recently burned soils [9, 10], a subset of 39 viromes was prepared for library construction and sequencing.

### Total DNA extraction and quantification

For each sample, 0.25 g of soil was added directly to the DNeasy PowerSoil Pro kit (Qiagen) for total DNA extraction, following the manufacturer’s protocol, with the addition of 10 min incubation at 65°C prior to cell lysis. DNA quantification was performed using the Qubit dsDNA HS Assay and Qubit 4 fluorometer (ThermoFisher).

#### Virome library preparation and sequencing

Sequencing of all 39 viromes was performed by the DNA Technologies and Expression Analysis Core at the University of California, Davis Genome Center (Davis, CA, USA). Metagenomic libraries were constructed with the DNA Hyper Prep kit (Kapa Biosystems-Roche, Basel, Switzerland) and sequenced (paired-end, 150 bp) using the NovaSeq S4 platform (Illumina, San Diego, CA, USA) to a requested depth of 10 Gbp per virome.

#### Amplicon library preparation and sequencing

We performed 16S rRNA gene amplicon sequencing on all total DNA extractions with a dual-indexing strategy [30]. To amplify the V4 region of the 16S rRNA gene, we used the Platinum Hot Start PCR Master Mix (ThermoFisher) with the 515F/806R universal primer set. We followed the Earth Microbiome Project’s PCR protocol [31], which included an initial denaturation step at 94°C for 3 min, 35 cycles of 94°C for 45 s, 50°C for 60 s, and 72°C for 90 s, and a final extension step at 72°C for 10 min. We cleaned libraries with AmpureXP magnetic beads (Beckman-Coulter), quantified amplicons with a Qubit 4 fluorometer (ThermoFisher), and then pooled all bead-purified products together in equimolar concentrations. The libraries were submitted to the DNA Technologies and Expression Analysis Core at the University of California, Davis Genome Center and sequenced (paired-end, 250 bp) using the MiSeq platform (Illumina).

#### Virome bioinformatic processing

Raw reads were trimmed using Trimmomatic v0.39 [32] with a minimum q-score of 30 and a minimum read length of 50 bases. We used BBMap v39.01 [33] to remove PhiX sequences. Each virome was assembled separately with MEGAHIT v1.2.9 [34] in the metalarge mode with a minimum contig length of 10 000 bp. Assembled contigs were analyzed with VIBRANT v1.2.0 [35] with the -virome flag to identify viral contigs. All output viral contigs (low, medium, and high quality) from all samples were iteratively clustered to form a representative set of vOTUs. We used CD-HIT v4.8.1 [36] to cluster viral contigs at 95% shared nucleotide identity and 85% alignment coverage (breadth). Quality-filtered raw reads from all viromes were then mapped against the representative set of vOTUs using Bowtie 2 v2.4.1 [37] in sensitive mode. Lastly, we used CoverM v0.5.0 [38] to quantify vOTU relative abundances in each virome and generated a trimmed mean coverage table and a count table for downstream analyses (0.75 minimum covered fraction). Host taxonomy of all vOTUs was predicted using iPHoP v1.1.0 [39], with default parameters (minimum cutoff score of 90) (Supplementary Table S4).

#### Virome read mapping to habitat-labeled vOTUs from other datasets

To investigate whether vOTUs in our dataset were previously found in other habitats, we mapped reads (with Bowtie 2 v2.4.1, as described before) to the PIGEON v2.0 vOTU database [40, 41], which contained 515 763 vOTUs, and two combined datasets collected from Blodgett Research Forest [10, 42], which contained 272 017 vOTUs. For the Blodgett datasets, we identified vOTUs that were only found in burned mixed conifer forest samples (labeled as soil - burned forest), and vOTUs that were only found in unburned mixed conifer forest samples orfound in both burned and unburned forest samples (labeled also as soil - forest). We then retrieved sequence data for all vOTUs that met minimum coverage (CoverM – 0.75 minimum covered fraction) by using the filterbyname tool from BBMap v39.01 on both the PIGEON v2.0 database and the Blodgett Forest combined datasets. We determined the proportion of vOTUs found in our dataset that were previously found in each of the other habitats. Finally, we clustered these vOTUs with the rest of the dataset at 95% average nucleotide identity (ANI) using CD-HIT [36], redid read mapping to this updated set of vOTUs, and generated a new trimmed mean coverage table for downstream analysis.

#### 16S rRNA gene amplicon sequence bioinformatic processing

We used DADA2 v1.12.1 [43] to demultiplex 16S rRNA gene amplicon reads and perform quality assessment, read merging, and chimera removal. For quality filtering, we used the filterAndTrim() function with the following parameters: truncLen = c(0,0), maxN = 0, maxEE = c(2,2), truncQ = 2, rm.phix = TRUE. All other processing was with default settings. Amplicon sequence variants (ASVs) were assigned taxonomy by using the DADA2 RDP classifier, using the SILVA database v138.1 as reference [44].

### Statistical and ecological analyses

R v4.4.1 was used to perform all ecological and statistical analyses [45]. Input for all analyses of viral communities or vOTUs was the trimmed mean coverage vOTU table. We normalized the vOTU abundance table by using the decostand function (method = “total”, MARGIN = 2) from the Vegan package v2.6–6.1 [46] and removed singletons (vOTUs detected in only one sample). With a DADA2-generated abundance table for 16S rRNA gene ASVs, we filtered out mitochondria and chloroplasts and removed singletons (ASVs that only appeared in a single sample). We calculated virome species richness via presence/absence in the normalized vOTU abundance table and prokaryotic species richness via presence/absence in the rarefied ASV abundance table. We calculated Bray-Curtis dissimilarities with vegdist (method = “bray”), performed PERMANOVAS with adonis2, and performed PERMDISPs with betadisper and permutest, all functions from the Vegan package. We performed multidimensional scaling and retrieved true eigenvalues to calculate PCoA points with the cmdscale function (base stats). To perform principal component analysis (PCA), we used the princomp function (base R). Upset pots were created with the ComplexUpset v1.3.3 package using species abundance tables that were transformed into presence-absence tables [47]. We determined distance between plots with the geosphere v1.5–18 package [48]. Statistical analyses are documented in Supplementary Tables S5–S7.

## Results and discussion

### Study design and dataset features

**Figure 1:**
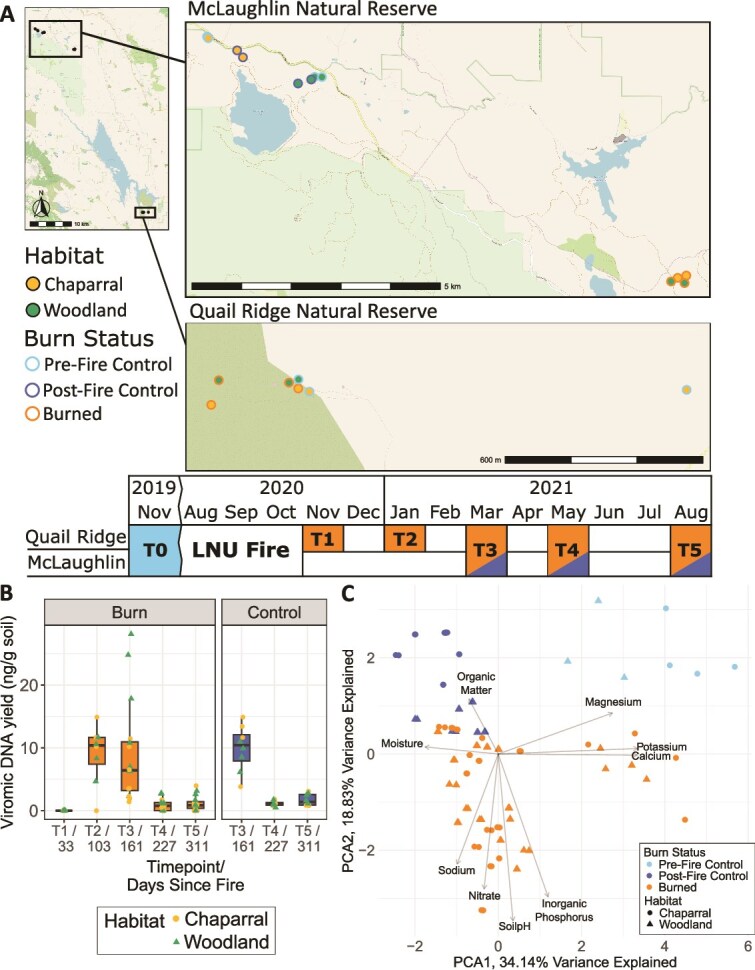
A. Map of plot locations in the main map and point fill indicating habitat and point outline indicating burn status in the subset maps, for a total of 12 plots. Two replicate cores were collected from each plot (each point in the map) at each sampled time point (except for the pre-fire controls). Table shows timeline of sample collection in each natural reserve. B. DNA yields for all viromes over time (labels show time point/days since fire), separated by burned (left facet) and control (right facet) plots. Dot color indicates habitat. Box boundaries correspond to 25th and 75th percentiles, and whiskers extend to ±1.5x the interquartile range. T1 and T2 were not collected for control plots but remain as “blanks” in the graph for easier comparisons to burned plots T3–T5. T0 (not plotted) was 322 days before the fire. C. PCA of z-transformed physicochemical properties for each sample, with color indicating burn status.

The intention for this study was to assess time-resolved changes in dsDNA viral communities after a wildfire, comparing the response in two habitats (chaparral and woodland) to control (not burned) plots (Fig. 1A). Over the course of 10 months (or 1 year since the start of the wildfire), we sampled five timepoints (T1–T5) at Quail Ridge Natural Reserve (CA) and three timepoints at McLaughlin Natural Reserve (CA) (corresponding with T3–T5 at Quail Ridge) for a total of 88 samples. For comparison, we also leveraged a pre-burn dataset of seven viromes from the same field sites collected in November 2019 [21] (here called “T0”, 9 months pre-burn).

We initially generated 88 DNase-treated viromes and used the viromic DNA yields to estimate patterns of viral particle abundances (as in prior work, [9]) and to determine which viromes to sequence (Fig. 1B). Yields were below detection limits for all samples at T1 (all of which were burned samples), indicating low viral particle abundances [9], consistent both with the dry season at the time of sampling and the potential long-term temperature and moisture impacts of the fire [49, 50], all of which have been shown to reduce viromic DNA yields [9, 10]. For example, viromic DNA yields from prior studies in dry grassland soils were below detection limits or at most 1.71 ng/g, compared to wet soils with yields ranging from 1.02 to 8.57 ng/g of soil [9], and yields were below detection limits in forest soils heated to 90°C [10]. For the three time points with control samples (T3–T5), there were significant differences in viromic DNA yields between burned and control samples for T3 and T4 in chaparral only (*P* < .05). There were no significant differences at any of the time points for woodland soils. This suggests a return to baseline viral particle abundances within 5 months of the fire (the first control timepoint) for woodland soils, and within 10 months of the fire (the third control timepoint) for chaparral soils. By T4 and T5, viromic DNA yields had decreased significantly in both chaparral and woodland habitats in both control and burned samples (Kruskal–Wallis, *P* < .05 for all conditions). Given that soils from T4 and T5 had significantly lower moisture contents than those from T2 and T3 (*P* < .05 for all pairwise comparisons), with T5 moisture content indistinguishable from T0 soils collected at the end of the 2019 dry season (Supplementary Fig. S1A), presumably, the observed decrease in viromic DNA yields reflects the transition into the dry season in the Mediterranean climate, which has previously been shown to substantially reduce the abundance of free viral particles [9].

Due to undetectable and low DNA yields, we elected not to sequence 49 of the viromes, which, to preserve statistical power for comparisons, reduced the number of time points considered for our viral community analysis. We sequenced 39 viromes, including all viromes from Quail Ridge (burned) from T2-T4 except one (Chaparral, T2) and all viromes from McLaughlin T3, which included the control viromes. We also extracted total DNA from all 88 samples and amplified the 16S rRNA gene region to profile prokaryotic communities. All 88 samples were amplified and sequenced, but one sample yielded insufficient sequencing data (Quail Ridge, Chaparral, T5), so it was removed from the analysis during rarefaction. We recovered 5097 ASVs over the entire dataset (Supplementary Table S8), which reduced to 4933 ASVs after rarefying to a common depth of 17 700 reads per sample and removing singletons.

The additional viromes that were collected in November 2019 [21] served as pre-fire controls and increased the number of vOTUs used for read mapping-based vOTU detection in our dataset. Paired 16S rRNA gene amplicon profiles were planned for that dataset, but, due to the COVID shutdown, they were never generated, and the soils are no longer available. In total, we amassed 77 869 putative viral contigs >10 bp as identified by VIBRANT [35], 77 433 of which were de novo assembled from this study, and 436 of which were vOTUs from the 2019 viromes. We also mapped reads to vOTUs from the PIGEON v2.0 database [41] and from viromes generated from our recent prescribed burn study in a forest [42], resulting in detection of 1644 additional vOTUs. All recovered viral contigs and vOTUs were then clustered into a dereplicated set of 71 046 vOTUs (Supplementary Table S9), which was reduced to 37 926 vOTUs after removing singletons (vOTUs detected in only one sample).

### Viral communities differed significantly in burned and unburned samples and between habitats, whereas habitat-distinct prokaryotic communities became more similar in burned soils

**Figure 2:**
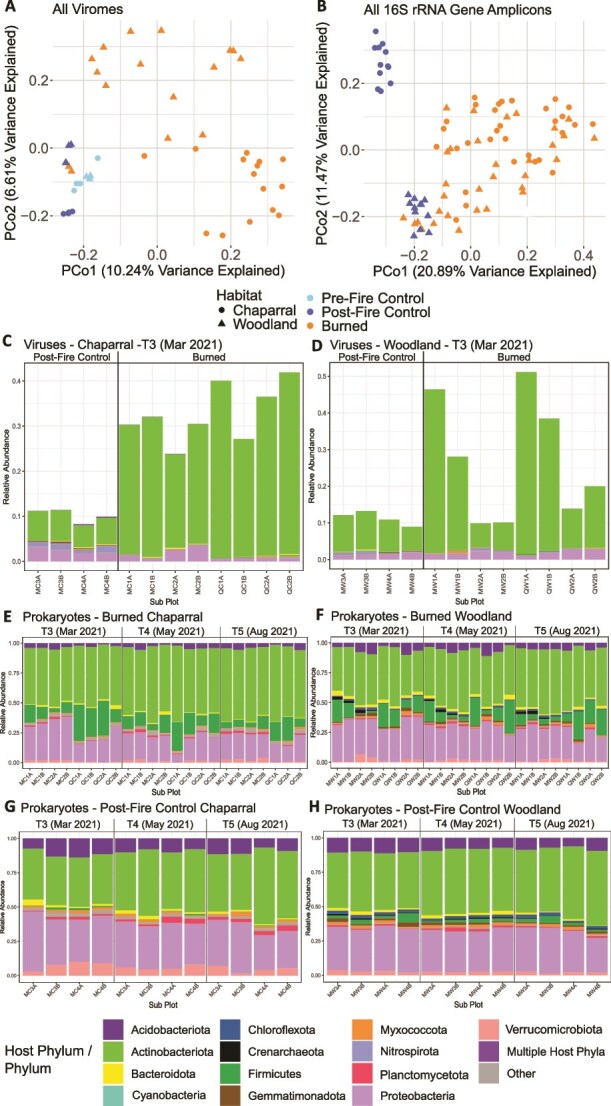
A/B. Unconstrained analyses of principal coordinates (PCoA) performed on vOTU (A) and prokaryotic ASV (B) Bray-Curtis dissimilarities calculated across all viromes or ASV gene profiles (from 16S rRNA gene amplicon data), respectively, with color indicating burn condition and shape indicating habitat. C/D. Relative abundances of vOTUs labeled with their phylum-level host predictions for chaparral (C) and woodland (D) habitats. Each stacked bar is one virome from a particular subplot. Less abundant host phyla are collapsed into the “other” group. E–H. Phylum-level relative abundances in 16S rRNA gene profiles from total DNA amplicon libraries of all samples over time, faceted by chaparral burned (E), woodland burned (F), chaparral post-fire control (G), and woodland post-fire control (H). Each stacked bar is one sample from a particular subplot, and all other parameters are like C/D. For C–H, subplot labels consist of time point (T1–T5) if applicable, field site (M for McLaughlin or Q for Quail Ridge), habitat (C for chaparral, W for woodland), plot number (1–4), and replicate (A or B). Legend colors are for C–H, with the “mixed” category only referring to C/D, where predicted hosts came from multiple phyla.

Since soil chemical properties are a good indicator of fire impact [6], we first compared the profile of chemical properties across soil samples. A PCA revealed significant separation of burned samples from post-fire controls based on soil chemistry (*P* = .04 by PERMANOVA) (Fig. 1C). The loadings in the direction of the burned samples relate to post-fire responses reported elsewhere, including increased pH [51] and increased available phosphorus [6, 11]. We then compared beta-diversity of viral and prokaryotic communities in post-fire and control samples to see whether they differed significantly. Burn condition (burned or unburned) significantly structured viral communities (explaining 13.1% of the variation, *P* < .001 by PERMANOVA) (Fig. 2A). Habitat explained 5.82% of the variation (*P* < .001 by PERMANOVA), still playing a part in viral community structuring, as has been observed previously [21]. Despite large spatial differences in sampling locations, spatial distance (plot location) did not seem to obscure treatment effects on viral communities as substantially as has been observed in other studies [28, 52], including in our analysis of viral community responses to a prescribed burn in a California mixed conifer forest [42] (*P* < .001 by PERMANOVA for plot location across all viromes, and *P* < .001 across all prokaryotic profiles). This means that plots with the same condition (burned or unburned) or the same habitat were most similar, overcoming plot-specific variability. While we did see viral community compositional structuring by habitat in control samples, as in prior work [21], habitat had a more pronounced influence on viral community composition post-fire (interaction of habitat and burn condition explained 8.1% of the variation in the data, *P* < .001 by PERMANOVA). The dispersion of viral communities was significantly greater in burned viromes than in controls (*P* = .006 by PERMDISP), meaning that burned viral community composition had greater variability than in unburned controls. We then considered vOTU detection patterns in each habitat (Supplementary Fig. S1B, see Supplementary Fig. S1C for comparison to prokaryotic ASV detection patterns, described below). With regard to soil chemical properties among the three different burn-status groups (Pre-Fire Control, Post-Fire Controls, and Burned), burned samples were significantly more dissimilar than post-fire controls (Tukey’s HSD, *P*.adj < .05). There were no significant dispersion differences between habitats. Of the 37 926 total number of vOTUs, 30% were detected only in post-fire burned woodland and 13% only in post-fire control woodland. For chaparral, 23% were found only in post-fire burned locations and 11% only in post-fire control locations. Only 11% of vOTUs were found in both burned habitats and 1.3% in both control habitats. Given this low percentage of shared vOTUs across habitats and/or burn conditions, these results indicate that habitat- and burn-specific vOTUs contributed more to community differences in the burned habitats, rather than shifts in the relative abundances of vOTUs shared across habitats and/or burn conditions. With regard to vOTU species richness, there were no significant differences between burned and post-fire control samples for either habitat (Supplementary Fig. S2A). However, there was a significant difference in richness between pre-fire control samples and both burned and post-fire control samples (*P* < .05, Dunn’s test), with pre-fire control samples yielding significantly fewer vOTUs. This is likely because the pre-fire control samples were taken in November when soil conditions were dry, whereas the other samples were collected in January, March, and May, which all had higher soil moisture, even in burned sites (Supplementary Fig. S1A).

For prokaryotic communities, burn condition explained 12% of the variation, habitat explained 8.2%, and the interaction between burn condition and habitat explained 5.6% (all *P* < .001 by PERMANOVA) (Fig. 2B). In terms of dispersion, prokaryotic communities in woodland and chaparral were more similar to each other in burned samples than control samples, despite habitat differences (*P* = .002 by PERMDISP). This suggests environmental filtering from the fire (e.g. due to changed physicochemical characteristics selecting for specific communities and/or differential survival after the fire) as a control on prokaryotic community assembly post-fire, as was also observed in a previous study of post-wildfire surface soils in a conifer forest [53]. Given the distinct separation between control and burned samples in soil chemical properties, prokaryotic, and viral communities across space, time, and habitat, this wildfire likely had a more uniform impact on the landscape than did a prescribed burn in our previous study of a forest habitat [42], likely attributable to overall greater fire severity. While heterogeneous burn severity over short distances best explained community compositional responses to fire in the previous study, with low-severity burned communities appearing similar to unburned controls and generally only high-severity communities exhibiting a significant response to burn, here the response was more consistent across burned samples. Of the 4933 recovered ASVs, the largest group of ASVs (18%) was found in both burned habitats, with an additional 12% detected in all sites, regardless of burn status. Within habitats, 8% of ASVs were found in both burned and unburned woodland sites and 6% in both burned and unburned chaparral sites. In contrast with viral communities, these results show that prokaryotic communities shared a greater proportion of ASVs post-fire, regardless of habitat (Supplementary Fig. S1C). In terms of ASV species richness, there were no significant differences for either habitat between burned samples and post-fire control samples (Supplementary Fig. S2B).

### Taxonomic shifts in burned versus control samples differed by habitat

To assess whether viral infection dynamics may have changed after wildfire, we next compared viromes according to predicted host taxonomic profiles between burned and unburned samples, leveraging data from T3 (March 2021, ~5 months since the fire), from which we could make these direct comparisons (due to low DNA yields, an insufficient number of viromes was sequenced from other time points). In chaparral, there was a significant increase in the relative abundances of phages predicted to infect Actinobacteria after wildfire and significant decreases in the relative abundances of putative phages of Acidobacteriota and Nitrospirota (Kruskal–Wallis, *P* < .05) (Fig. 2C). The woodland results were less consistent, with some comparisons showing an increase in putative phages of Actinobacteria, but not uniformly across burned samples. Only the relative abundances of viruses predicted to infect Cyanobacteria had a significant change (Kruskal–Wallis, *P* < .05) between burned and control viromes in the woodland habitat (Fig. 2D).

We were able to compare the relative abundances of prokaryotic phyla in control and burned samples for all three timepoints from which control samples were collected (T3–T5, Fig. 2E–H). In chaparral specifically, multiple prokaryotic phyla remained significantly different in burned samples as compared to control samples until T5 (August 2021, ~1 year since the fire), with some significantly different between burned and control samples across all timepoints (Figs. 2E and G). The relative abundances of Actinobacteriota and Firmicutes were significantly higher in burned samples at each timepoint, perhaps reflective of the ability of members of these phyla to form spores as survival structures [54, 55] and/or attributable to their abilities to use different carbon sources at different temperatures [56]. Increased relative abundances of Firmicutes have been previously observed in the first year after a wildfire in chaparral [12] and forest ecosystems [57], as well as in heated soil [10]. Although there was a significant shift in the relative abundances of viruses predicted to infect Nitrospirota, a phylum of nitrite-oxidizing bacteria that typically dominates in arid ecosystems [58], members of this phylum were at very low relative abundance in the prokaryotic data and did not exhibit the same pattern as their viruses. In woodland habitats (Figs. 2F and H), similar to the patterns in the viruses when grouped by predicted hosts, differences in prokaryotic communities between burned and control samples were less pronounced and were observed at fewer time points. Firmicutes were significantly more abundant in burned samples (only at T4 and T5), similar to our observations in chaparral (Kruskal–Wallis, *P* < .05). In general, the woodland habitat did not exhibit as pronounced a response to the fire, in terms of phylum-level shifts in the relative abundances of prokaryotes or their predicted phages. We investigated a few of the genera considered to be pyrophilous [12, 16–20]. The relative abundances of *Massilia*, in the phylum Proteobacteria, were significantly higher in every timepoint for both habitats (Supplementary Fig. S2C and D). The relative abundances of *Bacillus*, in the phylum Firmicutes, were also significantly higher in every timepoint for both habitats, with the exception of T3 in the woodland habitat (Supplementary Fig. S2E and F).

Of the three timepoints that had both burned and unburned samples (T3–T5), only one timepoint had sufficient data to compare viral community compositional shifts between the two sample types. Thus, we were unable to track changes over time in burned and unburned soils. However, we were able to determine whether there were significant compositional changes for viruses (based on host-prediction data) or prokaryotes in burned soil as the days since fire increased. In chaparral, there were significant changes in the relative abundance of viruses predicted to infect Actinobacteria and Bacteroidota (decreasing and increasing over time, respectively) (Supplementary Fig. S3A), while in woodland, putative viruses of Bacteroidota and Proteobacteria significantly decreased over time (Spearman’s, *P* < .05) (Supplementary Fig. S3B). Viruses predicted to infect all other phyla with significant changes in their relative abundances across timepoints (Kruskal–Wallis, *P* < .05) had trends that were non-monotonic (meaning, not consistently increasing or decreasing) over time. For prokaryotes, most phyla that significantly differed in relative abundance across timepoints (Kruskal–Wallis, *P* < .05) also had non-monotonic relationships with days since fire, potentially reflecting seasonal changes (Supplementary Fig. S3C). Only Actinobacteria significantly increased over time in both chaparral and woodland habitats, and Acidobacteriota decreased over time in woodland (Spearman’s, *P* < .05).

Additionally, we sought to link soil chemical properties with viral and prokaryotic communities. A correlation analysis of the relative abundances of viruses grouped by phylum-level host predictions and soil chemical properties across all samples (including controls) revealed two dominant groups of viruses (Supplementary Fig. S4A). The first group, consisting of viruses predicted to infect Proteobacteria, Acidobacteriota, Nitrospirota, and Verrucomicrobiota, had significant negative correlations with soil chemical properties known to increase post-fire, decreasing in relative abundance with increases in soil pH, inorganic phosphorus, and potassium. This group of viruses also consistently significantly decreased in relative abundance with decreasing soil moisture. The second group, which notably included viruses predicted to infect Firmicutes and Actinobacteriota (known fire-responsive and potentially spore-forming taxa [54, 55]), generally responded more positively to fire-responsive changes in chemical properties. However, trends were inconsistent and mostly not significant, with only viruses of Actinobacteriota significantly increasing with increasing soil pH, nitrate, inorganic phosphorus, and sodium concentrations, and with decreasing soil moisture. When considering host taxa (at the phylum level to match the viral predictions), two distinct groups also emerged (Supplementary Fig. S4B). The first group included only Actinobacteriota and Firmicutes, with Firmicutes having significant positive correlations with almost all fire-responsive soil chemical properties. The second group of prokaryotes generally negatively correlated with fire-responsive soil chemical properties, though this was only consistently the case for Proteobacteria and Verrucomicrobiota.

### Biogeographic context suggests that some viral taxa were preferentially detected in post-fire datasets and thus may be fire-adapted

**Figure 3:**
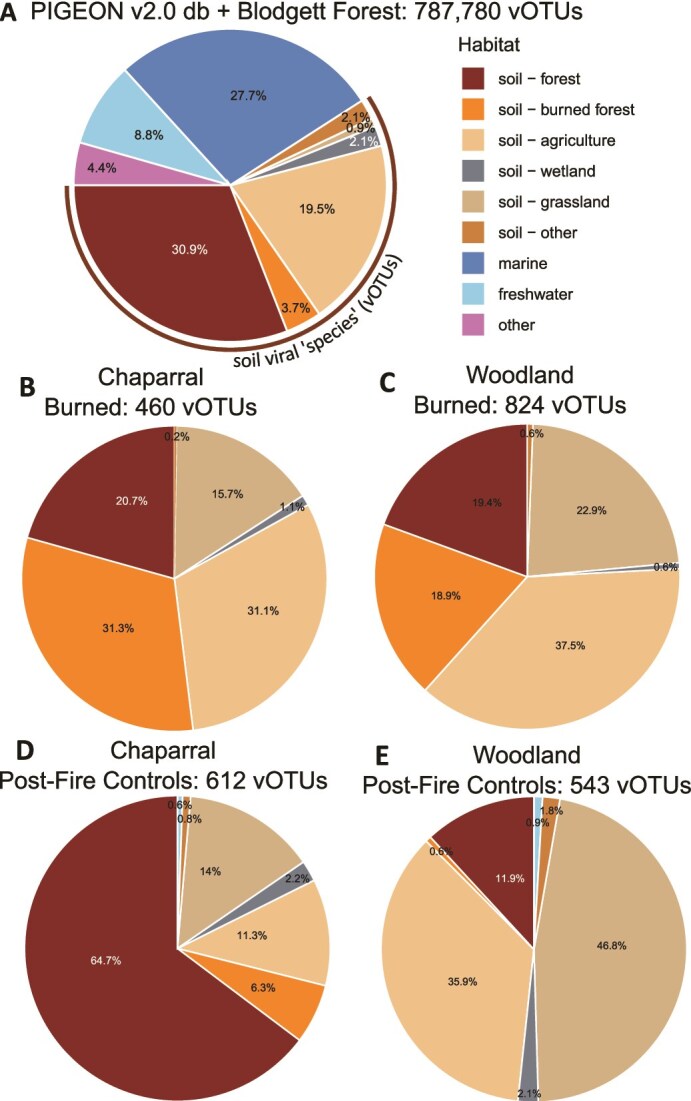
A. Habitat composition (percentages) of 787 780 vOTUS from the combined set of vOTUs from the PIGEON v2.0 database (db) and the Blodgett Forest prescribed burn and laboratory heating studies [10, 42], colored by original source environment. B–E. Relative proportions of the vOTUs from (A) that were detected in our dataset, separated by the habitat (chaparral or woodland) and burn status (burn or control) in which they were detected.

To assess biogeographic patterns of fire-impacted vOTUs, we performed read mapping of this LNU viromic dataset to other vOTU databases to see whether our viromes included vOTUs that had been previously detected elsewhere, including in our other fire-related studies. We detected a total of 1644 such vOTUs, with 760 from a mixed conifer forest soil as part of our prescribed burn study in Blodgett Forest, CA [10, 42] and 884 from the PIGEON v2.0 database [41]. The overall composition of vOTUs in the PIGEON v2.0 database together with the Blodgett Forest vOTUs reflected primarily aqueous (freshwater and marine), forest, and agricultural sources (Fig. 3A). Other than vOTUs from our November 2019 viromes from these sites, which we had already included in this study (and therefore removed from the database, leaving a total of 787 363 vOTUs), the PIGEON v2.0 database did not contain any vOTUs known to originate from chaparral or woodland habitats, though some vOTUs were from samples labeled as “terrestrial (soil)” in the original publication [59], which could conceivably have included these habitats. When separated by habitat and burn condition, a large percentage of vOTUs detected in burned chaparral (total vOTUs = 460) (Fig. 3B) and burned woodland (total vOTUs = 824) (Fig. 3C) viromes were originally found in burned mixed conifer forest (31.3% and 18.9%, respectively). Conversely, when considering the composition of vOTUs found in the control viromes (612 total vOTUs for chaparral and 543 total vOTUs for woodland), burned mixed conifer forest was a much smaller fraction (6.3% for chaparral, Fig. 3D, to almost undetectable at 0.6% for woodland, Fig. 3E). In burned chaparral, we were able to predict a host for 38 (26.4%) of the 144 vOTUs also detected in burned mixed conifer forest, with 34 vOTUs predicted to infect members of Actinobacteriota, two predicted to infect Proteobacteria, and two predicted to infect Firmicutes. In burned woodland, hosts were predicted for 71 (45.5%) of the 156 vOTUs also detected in burned mixed conifer forest, with 68 vOTUs predicted to infect Actinobacteriota and three predicted to infect Firmicutes. As these predicted hosts are also known fire-responsive taxa [17, 20], these findings suggest that there may be specific groups of viral “species” that commonly persist in post-fire environments, presumably in part due to survival and/or rapid colonization and growth of their hosts [9], but also perhaps due to their own inherent survival traits (e.g. as genomes inside spore-forming hosts and/or as durable virions) [60, 61].

## Conclusion

In this study, we compared viral community responses to wildfire in chaparral and woodland habitats over 1 year. Many of our viromes had low-to-undetectable viromic DNA yields, suggesting that post-fire conditions in arid climates likely have low extracellular viral biomass. Of the 39 viromes that we were able to sequence, significant differences were observed between burned and control viromes for both habitats, alongside significant changes in soil chemical properties and prokaryotic community composition. Wildfire increased dispersion in viral community beta-diversity for both habitats, while there was an opposite effect on prokaryotic community composition, with prokaryotic communities from different habitats more similar in burned samples than in controls. At T3 (the only timepoint from which control viromes were sequenced), significant increases in the relative abundances of viruses predicted to infect Actinobacteriota and decreases in putative Nitrospirota and Acidobacteriota phages were evident in chaparral, while only viruses predicted to infect Cyanobacteria significantly decreased in woodland. As for prokaryotic shifts, only Actinobacteriota and Firmicutes were consistently significantly more abundant in burned chaparral samples at all timepoints than the controls (suggesting increased survival of spore-formers), with no consistent changes in woodland. Additionally, there seemed to be a degree of environmental filtering on viral “species” in burned ecosystems, regardless of habitat, given that a substantial percentage of vOTUs in burned chaparral and woodland were also found previously in burned forest.

In summary, wildfire had a clear impact on viral community composition, including changes in the relative abundances of viruses predicted to infect bacteria from spore-forming phyla, which were at higher abundance in burned samples. The shifts in viral community composition post-fire were at least partially attributable to shifts in their host abundances, but perhaps some of these viruses were also inherently more durable (fire-resistant). Higher temporal resolution in both burned and unburned viromic data will help us to better characterize these compositional shifts over time and compare them with prokaryotic community shifts, enhancing our understanding of virus-host dynamics post-fire.

## Supplementary Material

Geonczy_SupplementaryFigures_ISMEComms_Mar2025_ycaf073

Geonczy_Wildfire_Supplementary_Tables_ISMEComms_Mar2025_ycaf073

## Data Availability

All raw sequences have been deposited in the NCBI Sequence Read Archive under the BioProject accession PRJNA1147726. The database of dereplicated vOTUs is available at https://zenodo.org/records/12740558. All scripts are available at https://github.com/seugeo/wildfire.
