# Supplementary material for: Soil viral communities shifted significantly after wildfire in chaparral and woodland habitats
Source: ISME Commun. 2025 May 6;5(1):ycaf073. doi: 10.1093/ismeco/ycaf073 (PMC12085916; doi:10.1093/ismeco/ycaf073)

Supplementary Figures and Captions

Soil viral communities shifted significantly after wildfire in chaparral and woodland habitats

Sara E. Geonczy, Anneliek M. ter Horst, Joanne B. Emerson

ISME Communications

Supplementary Figure 1. A. Gravimetric soil moisture content over time for sampled timepoints. Points correspond to individual samples, and trend lines represent the mean moisture content at each time point. Color indicates burn status. Gray shaded box outlined in orange represents timing of wildfire. **B/C.** UpSet plots of vOTU (**A**) and ASV (**B**) detection patterns across plots with blackened circles indicating detection in a particular habitat with a particular condition (burned, pre-fire control, and post-fire control for vOTUs, and burned and post-fire control for ASVs) and intersection size indicating the number of vOTUs or ASVs with a given detection pattern (detected in one or more habitat/burn condition combinations).

Supplementary Figure 2. A/B. Richness of vOTUs (**A**) and ASVs (**B**) for each burn status. Fill corresponds to habitat. **C-F**. Relative abundances at each timepoint of genus Massilia in chaparral sites (**C**) and woodland sites (**D**) and genus Bacillus in chaparral sites (**E**) and woodland sites (**F**). Fill corresponds to burn status. For **A-F**, box boundaries correspond to 25th and 75th percentiles, and whiskers extend to ±1.5x the interquartile range.

Supplementary Figure 3. A/B. Time series showing the relative abundances of vOTUs grouped by their phylum-level host predictions for burned chaparral (**A**) and burned woodland (**B**) time points. Each bar is a virome, and vertical black lines separate time points. All lower abundance vOTU groups (according to host phyla) are collapsed into an “other” group. **C.** Time series showing relative abundances of prokaryotic phyla, faceted by habitat (chaparral – left, woodland – right) from burned samples from all timepoints. Each bar is a sample, with the missing bar representing a sample that failed amplification and was not sequenced. Vertical black lines separate time points. All lower abundance phyla are collapsed into an “other” group. For **A-C**, sample IDs consist of time point (T1-T5), field site (M for McLaughlin or Q for Quail Ridge), habitat (C for chapparal, W for woodland), plot number (1-4), and replicate (A or B).

Suplementary Figure 4. A/B. Hierarchical clustering and heatmap visualizing the correlation analysis of soil properties and relative abundances of (**A**) vOTU groups with a given phylum-level host prediction or (**B**) prokaryotic phyla. Gradient of colors indicate Pearson's correlation coefficient. Negative correlations correspond to dark blue, and positive correlations correspond to dark red. Asterisks indicate a significant correlation ($p < 0.05$).

Supplementary Figure 1.

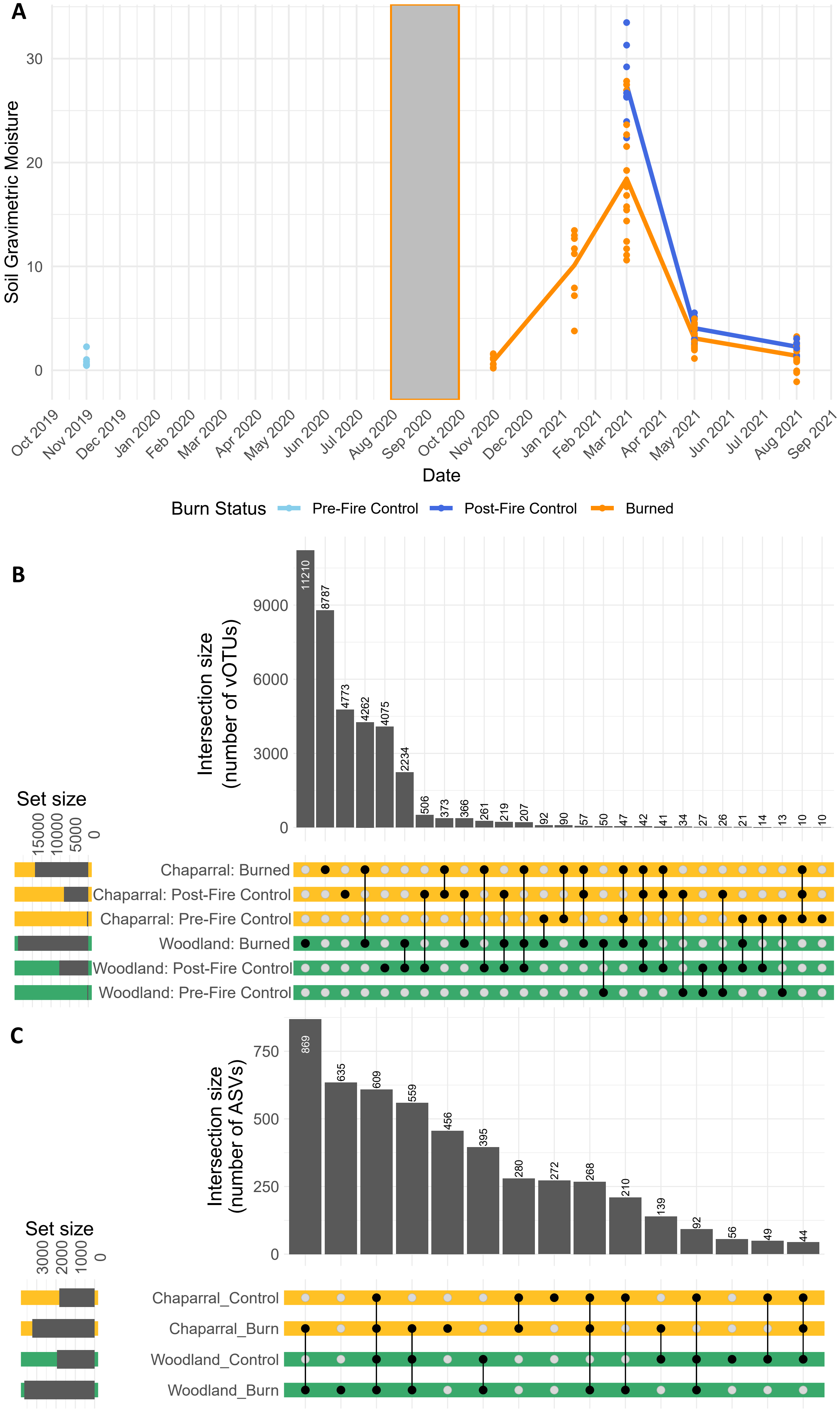

Supplementary Figure 2.

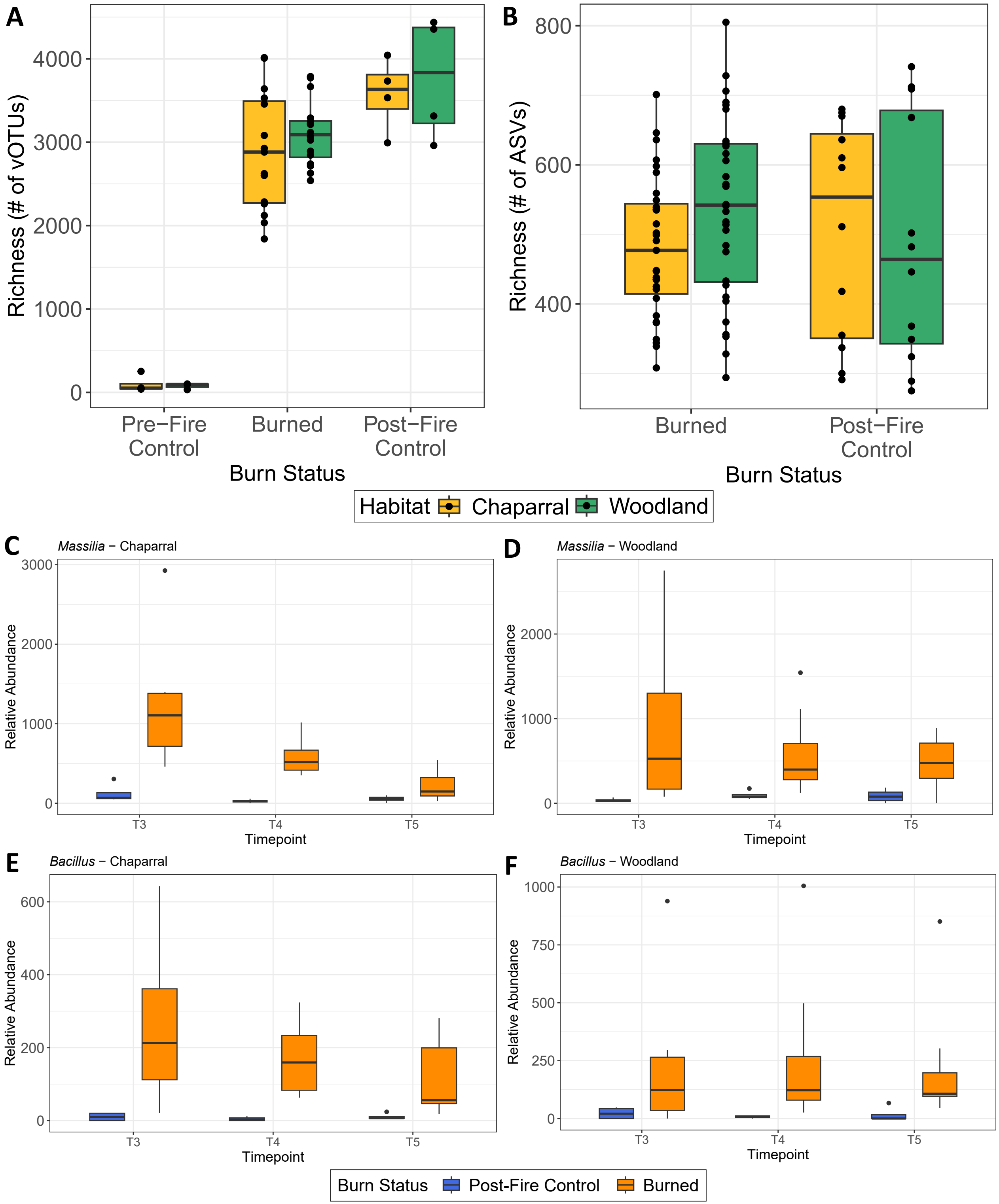

Supplementary Figure 3.

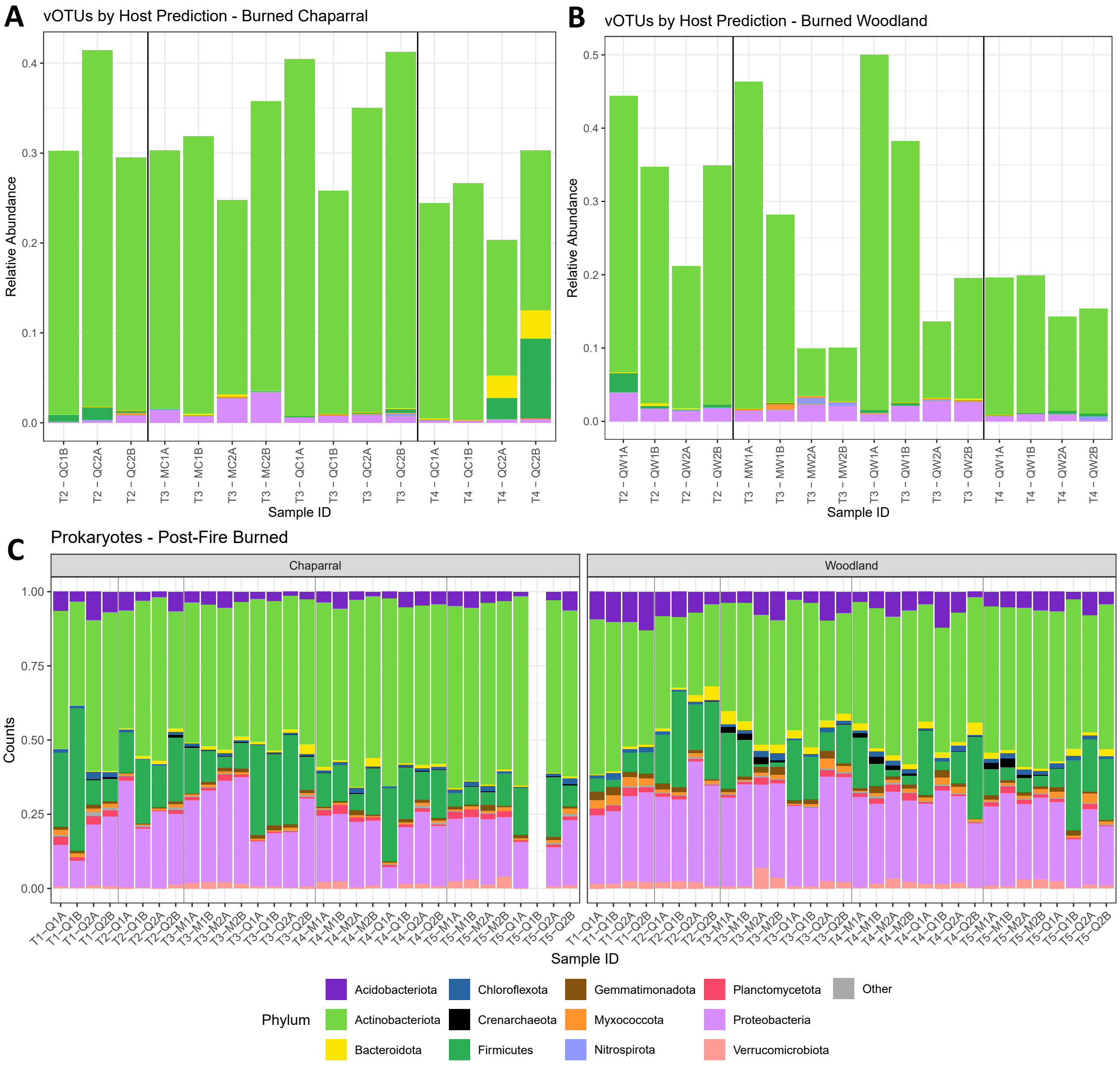

Supplementary Figure 4.

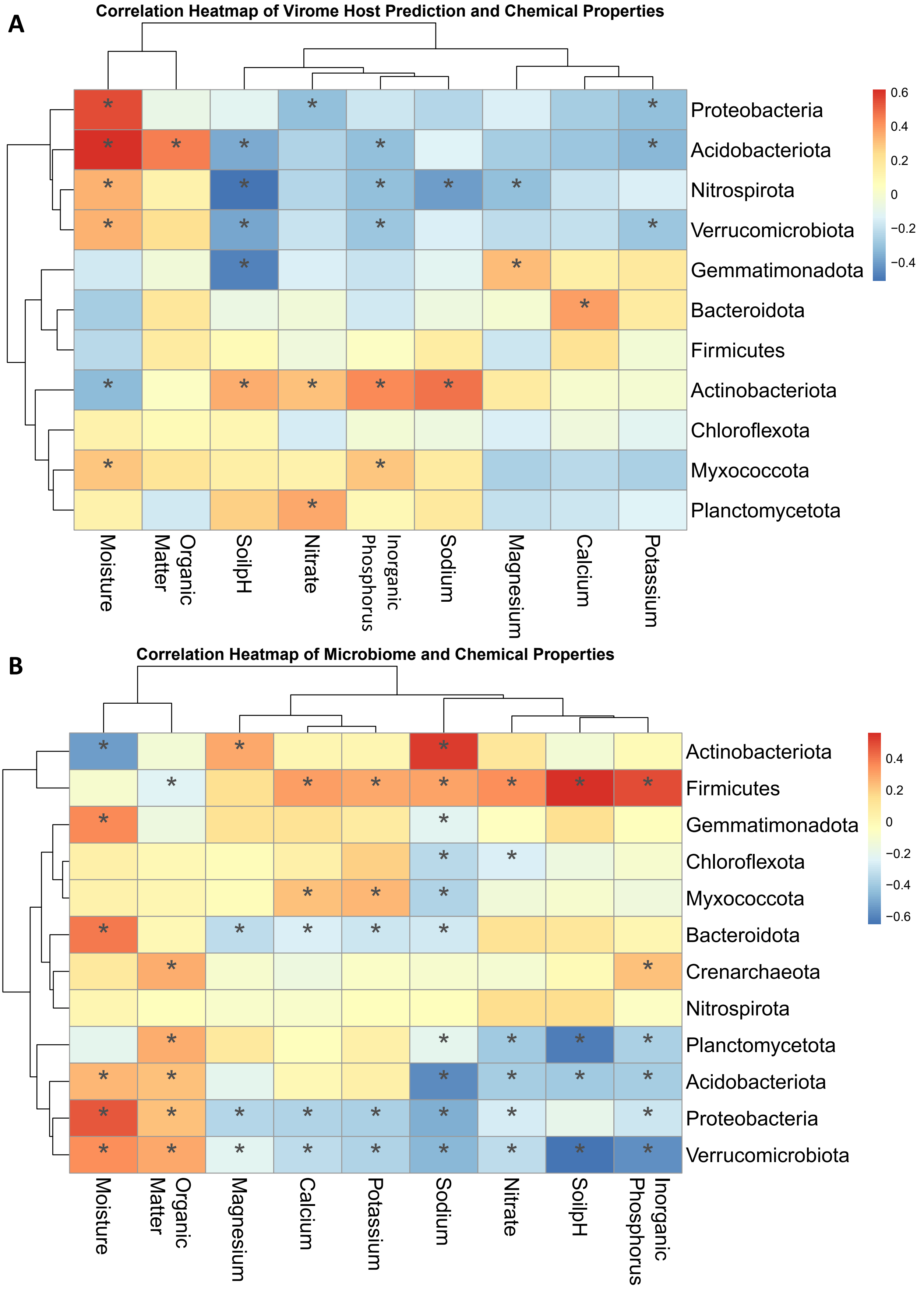

Supplement: Geonczy_SupplementaryFigures_ISMEComms_Mar2025_ycaf073 [file geonczy_supplementaryfigures_ismecomms_mar2025_ycaf073.pdf]
